# Medication for Opioid Use Disorder in Federally Qualified Health Centers

**DOI:** 10.1001/jamanetworkopen.2024.54772

**Published:** 2025-01-16

**Authors:** Zoe Lindenfeld, Amanda I. Mauri

**Affiliations:** 1 Edward J. Bloustein School of Planning and Public Policy, Rutgers University, New Brunswick, New Jersey; 2 Department of Public Health Policy and Management, School of Global Public Health, New York University, New York, New York

## Abstract

This cross-sectional study examines changes in and factors associated with rates of patients with substance use disorder receiving medication for opioid use at federally qualified health centers.

## Introduction

To reduce morbidity and mortality among individuals with opioid use disorder (OUD), the evidence is strongest for medications for OUD (MOUD), which include methadone, buprenorphine, and naltrexone.^1,2^ Federally qualified health centers (FQHCs) are essential in expanding access to MOUD, particularly buprenorphine, which can be prescribed in any health care setting.^3,4^ FQHCs service a high proportion of patients who have low income and are uninsured, populations disproportionately impacted by the overdose crisis.^5^ Despite the critical role of FQHCs in expanding access to MOUD, there is a scarcity of published research examining whether FQHCs prescribe MOUD to patients with OUD. We conducted a study to address these limitations by analyzing changes in the percentage of FQHC patients with substance use disorder (SUD) receiving MOUD, and identifying the FQHC characteristics associated with a higher proportion of patients receiving MOUD from 2017 to 2023.

## Methods

This cross-sectional study was deemed exempt from institutional review board approval and informed consent by Rutgers University because we used publicly available organizational-level data. This study is reported following the Strengthening the Reporting of Observational Studies in Epidemiology (STROBE) reporting guideline for cross-sectional studies.

We analyzed data on FQHCs in all 50 US states from 2017 to 2023, using the Uniform Data System (UDS), a publicly available dataset of health center operational information from the Health Resources and Services Administration. We extracted data on the total number of patients served, the number of patients with a SUD, the number of patients receiving MOUD (“How many patients received medication-assisted treatment for opioid use disorder from a physician, certified nurse practitioner, or physician assistant, with a DATA waiver working on behalf of the health center?”), the number of MOUD prescribers, demographic characteristics of FQHC patients (race, ethnicity, housing status, income, and HIV status), the state in which FQHCs are located, and whether the FQHC is located in an urban or rural area. Race and ethnicity were self-reported. We categorized race as non-White (ie, the percentage of patients identifying as American Indian/Alaskan Native; Asian; Black/African American; White, Hispanic/Latino; or Native Hawaiian or Pacific Islander) and White (ie, the percentage of patients identifying as White, non-Hispanic/Latino). We include this indicator to assess whether the racial composition of the FQHC population is associated with higher MOUD treatment receipt.

We present descriptive statistics for the number and percentage of patients with a SUD who received MOUD for each year (2017-2023). Additionally, we provide a longitudinal ordinary least-squares linear regression model with state-level clustering, accounting for within-observation correlation using mixed effects, to determine the association between the percentage of patients with SUD who receive MOUD and FQHC organizational and patient demographic characteristics. All measures were calculated at the FQHC level. *P* values were 2-sided, and statistical significance was set at *P* < .05. Analyses were conducted using Stata SE version 18 (StataCorp) from June to September 2024.

## Results

Across all years, 9800 observations were included in this analysis (1373 in 2017; 1362 in 2018; 1384 in 2019; 1375 in 2020; 1373 in 2021; 1370 in 2022; 1363 in 2023). The Figure displays the percentage of patients with an SUD who received MOUD each year, which increased over time from 10.01% in 2017 to 24.75% in 2023. Results from the regression (Table) demonstrate that the percentage of patients with an SUD receiving MOUD was positively and significantly associated with the percentage of patients who had an SUD (β = 0.77; 95% CI, 0.43 to 1.11) and the percentage of patients with HIV (β = 0.10; 95% CI, 0.00 to 0.21) and negatively and significantly associated with percentage of patients experiencing homelessness (β = −0.07; 95% CI, −0.13 to −0.01), and patients not identifying as White, non-Hispanic/Latino (β = −0.15; 95% CI, −0.22 to −0.08). Descriptive statistics of FQHC characteristics are also reported in the Table.

**Figure.**
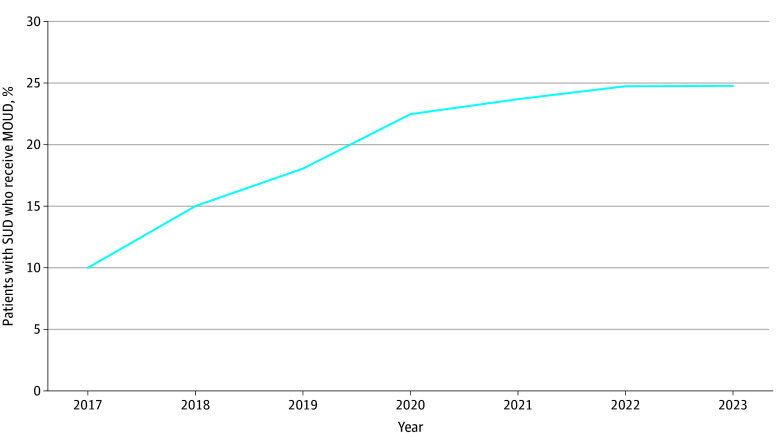
Percentage of Patients With Substance Use Disorder (SUD) Receiving Medications for Opioid Use Disorder (MOUD) by Year, 2017-2023

**Table.** Descriptive Statistics and Longitudinal Regression Results Estimating the Percentage of Patients With an SUD Receiving MOUD at FQHCs, 2017-2023 (N = 8992)

Variable	Overall mean (SD), %	β (95% CI)
Patients with an SUD	2.84 (4.63)	0.77 (0.43 to 1.11)^a^
Patients who are homeless	6.86 (17.24)	−0.07 (−0.13 to −0.12)^b^
Patients with HIV	7.65 (9.29)	0.10 (0.002 to 0.21)^b^
Patients best served in a language other than English	19.94 (22.95)	0.05 (−0.02 to 0.13)
Patients that are non-White^c^	59.56 (30.02)	−0.15 (−0.22 to −0.08)^a^
Patients with incomes ≤200% of the federal poverty level	62.28 (25.14)	−0.05 (−0.11 to 0.002)
FQHC located in an urban location, No.(%)	5513 (57.43)	0.16 (−3.78 to 4.11)
Total patients served, No.	21 452.85 (26 386.69)	4.09 × 10^−6^ (−3.43 × 10^−5^ to 4.24 × 10^−5^)
MOUD prescribers, No. per 1000 patients	0.41 (1.26)	NA

Abbreviations: FQHC, federally qualified health center; MOUD, medications for opioid use disorder; SUD, substance use disorder.

^a^ *P* < .01.

^b^ *P* < .05.

^c^ Patients identifying as American Indian/Alaskan Native; Asian; Black/African American; White, Hispanic/Latino; or Native Hawaiian or Pacific Islander.

## Discussion

This cross-sectional study found that while the percentage of patients with SUD receiving MOUD increased during our study period, in 2023, less than one-quarter of patients with SUDs at FQHCs received MOUD, signaling additional effort is needed to increase access to MOUD within FQHCs. Results from our regression model and the magnitude of our findings also indicate that efforts should target FQHCs serving a higher percentage of non-White patients and patients experiencing homelessness, populations that may face additional barriers to receiving MOUD.^6^ A limitation of this analysis pertains to our measure of patients with SUD taken from the UDS. While the UDS measure of SUD does not include alcohol use disorder or tobacco use disorder, it is not restricted solely to patients with OUD and may include patients with other SUDs. However, given our findings, even if 50% of patients with SUD qualified for MOUD, they would still likely be underserviced, as our results indicate that less than 25% of patients with SUD received MOUD in 2023. We were also unable to assess the type of MOUD prescribed at each FQHC because of limitations with the underlying data. Despite these limitations, our study underscores the significant opportunity to further increase MOUD prescribing in FQHCs. Policymakers at the federal, state, and FQHC level should prioritize opportunities to enhance MOUD access within FQHCs, for example, by providing staff education and training on MOUD; reviewing state policies, such as prior authorization, that may impede access to MOUD; and leveraging opioid settlement funds to support MOUD adoption.^5^

## Notes

Supplement.
Data Sharing Statement
