# Supplementary material for: Medication for Opioid Use Disorder in Federally Qualified Health Centers
Source: JAMA Netw Open. 2025 Jan 16;8(1):e2454772. doi: 10.1001/jamanetworkopen.2024.54772 (PMC11739986; doi:10.1001/jamanetworkopen.2024.54772)
Supplement: Supplement. — Data Sharing Statement [file jamanetwopen-e2454772-s001.pdf]

Data Sharing Statement

Lindenfeld. Medication for Opioid Use Disorder in Federally Qualified Health Centers. *JAMA Netw Open*. Published January 16, 2025. doi:10.1001/jamanetworkopen.2024.54772

Data
Data available: No

Additional Information
Explanation for why data not available: Data for this analysis is publicly available from the Human Resource Service Administration.
